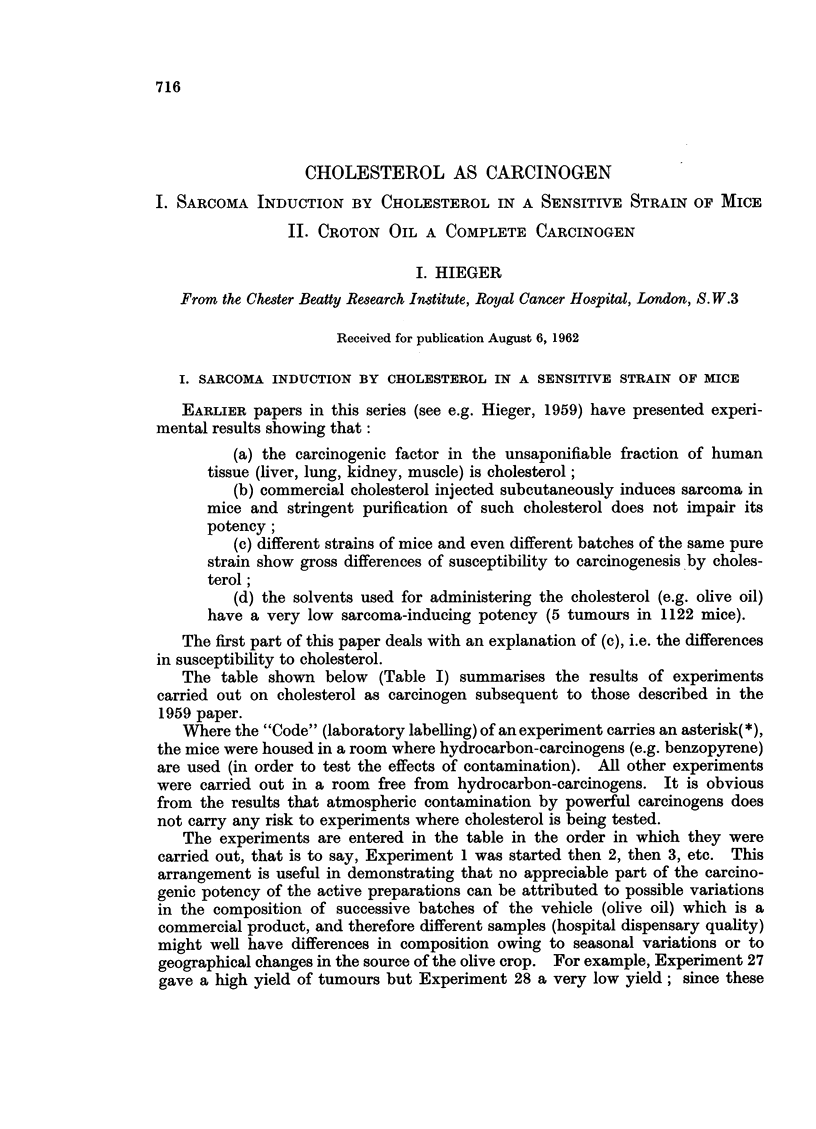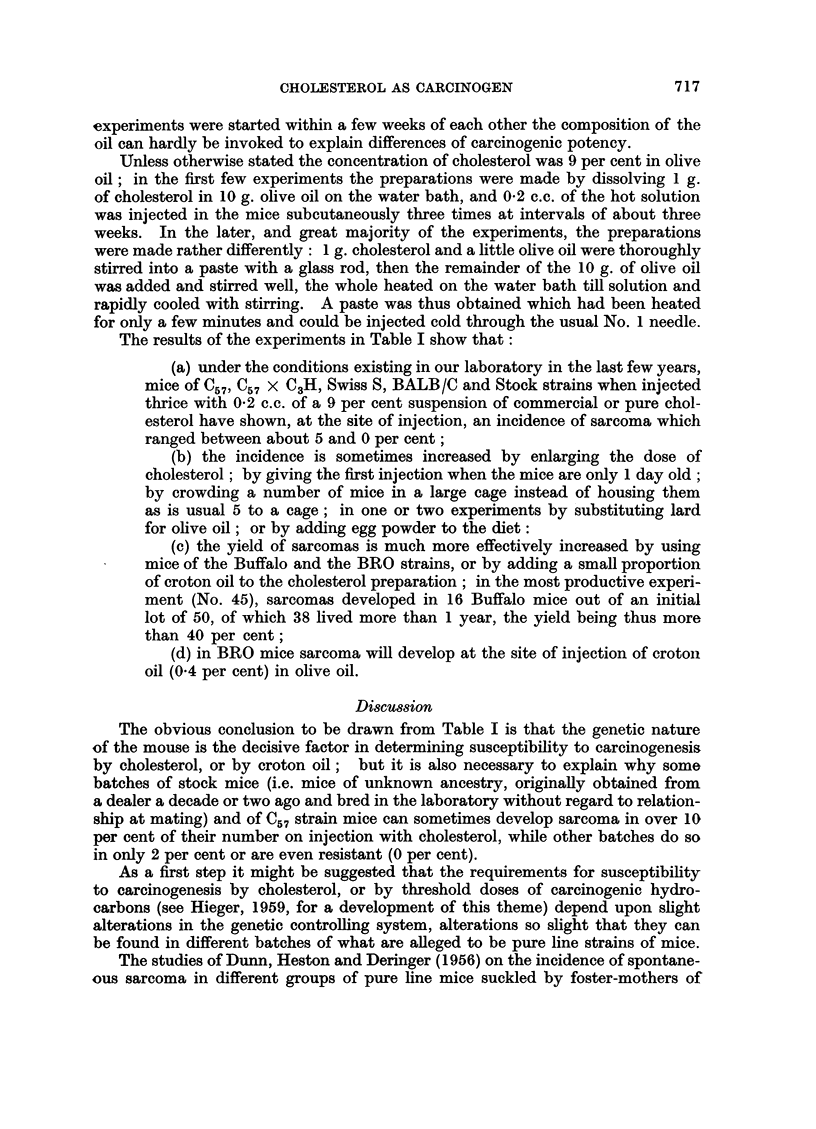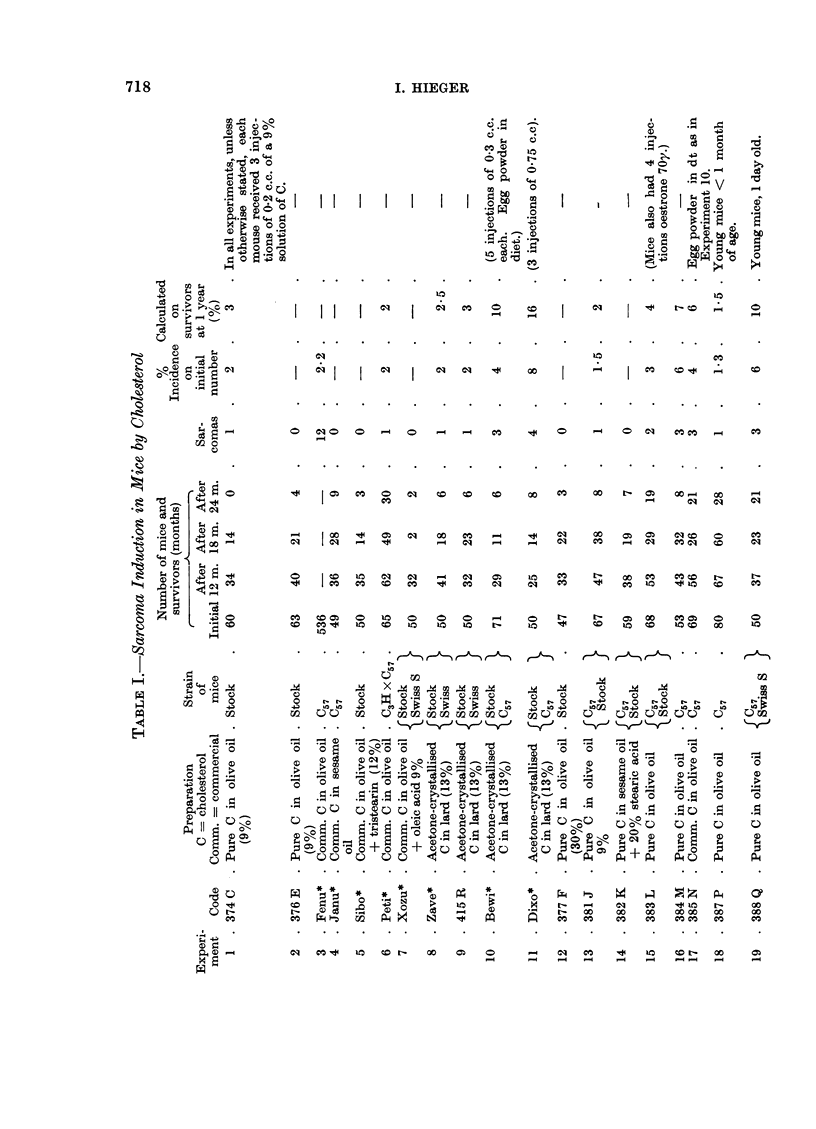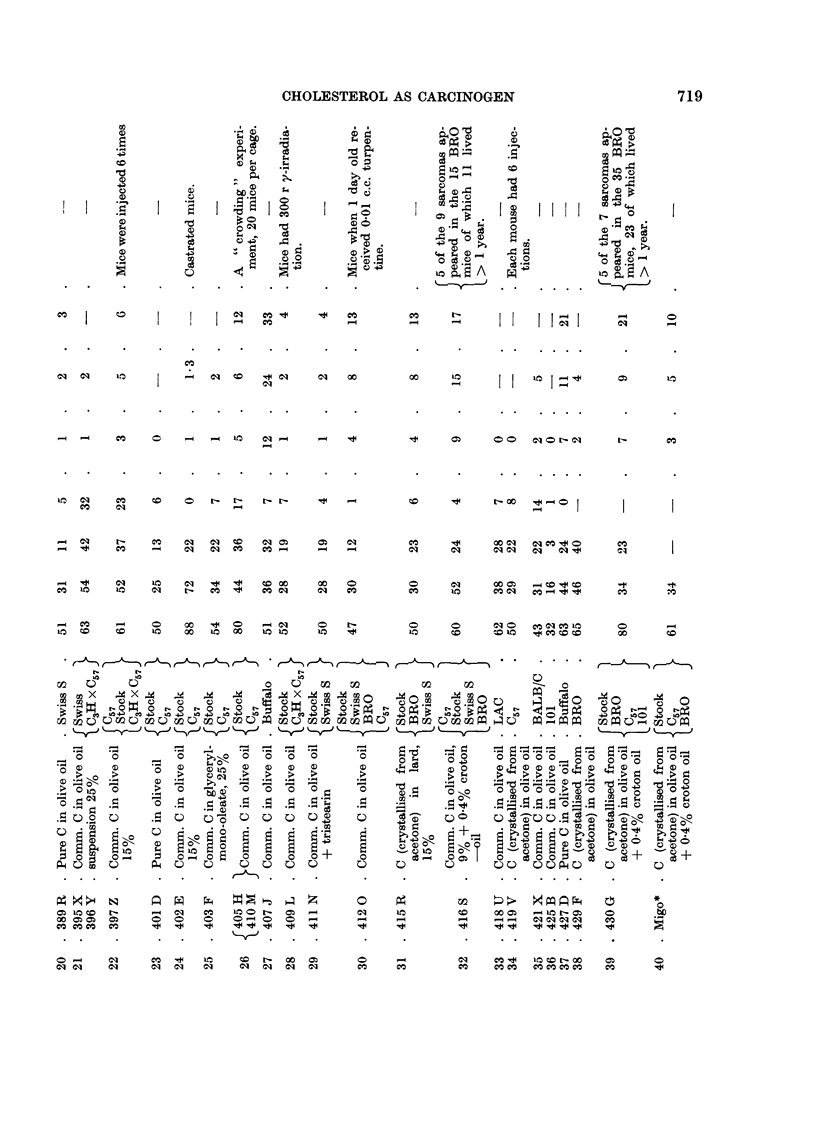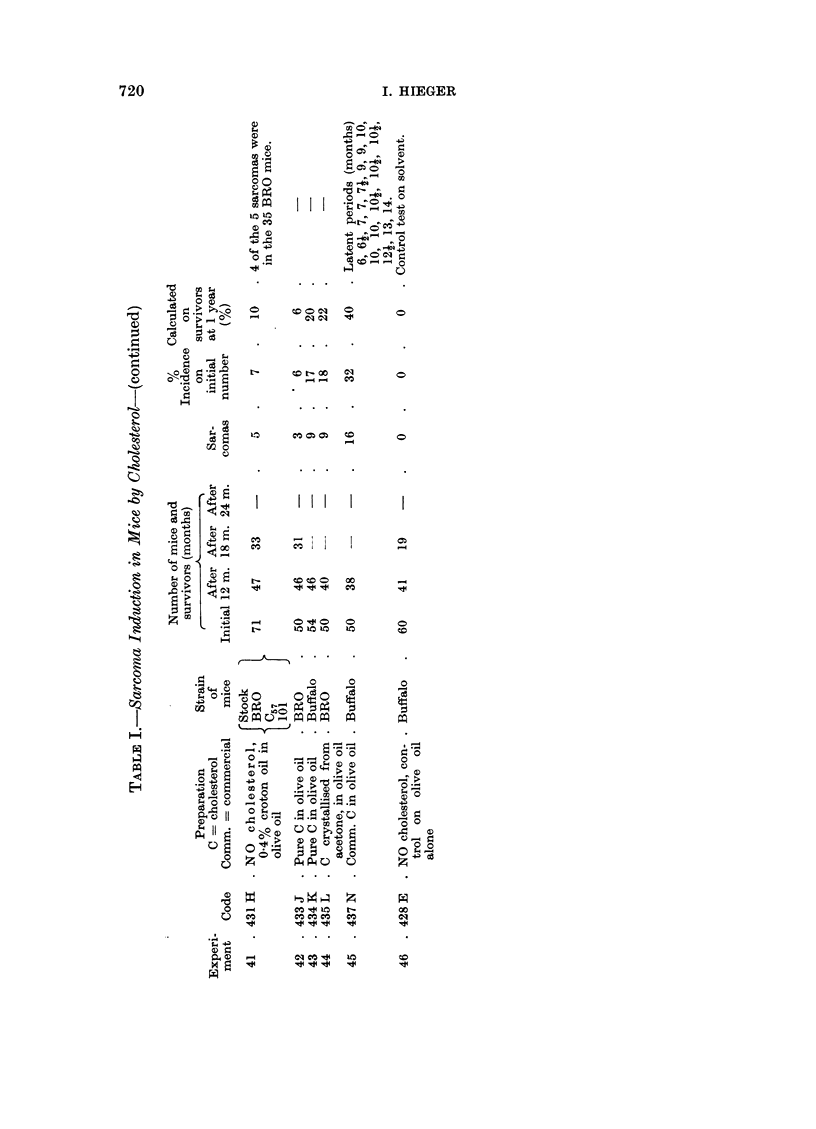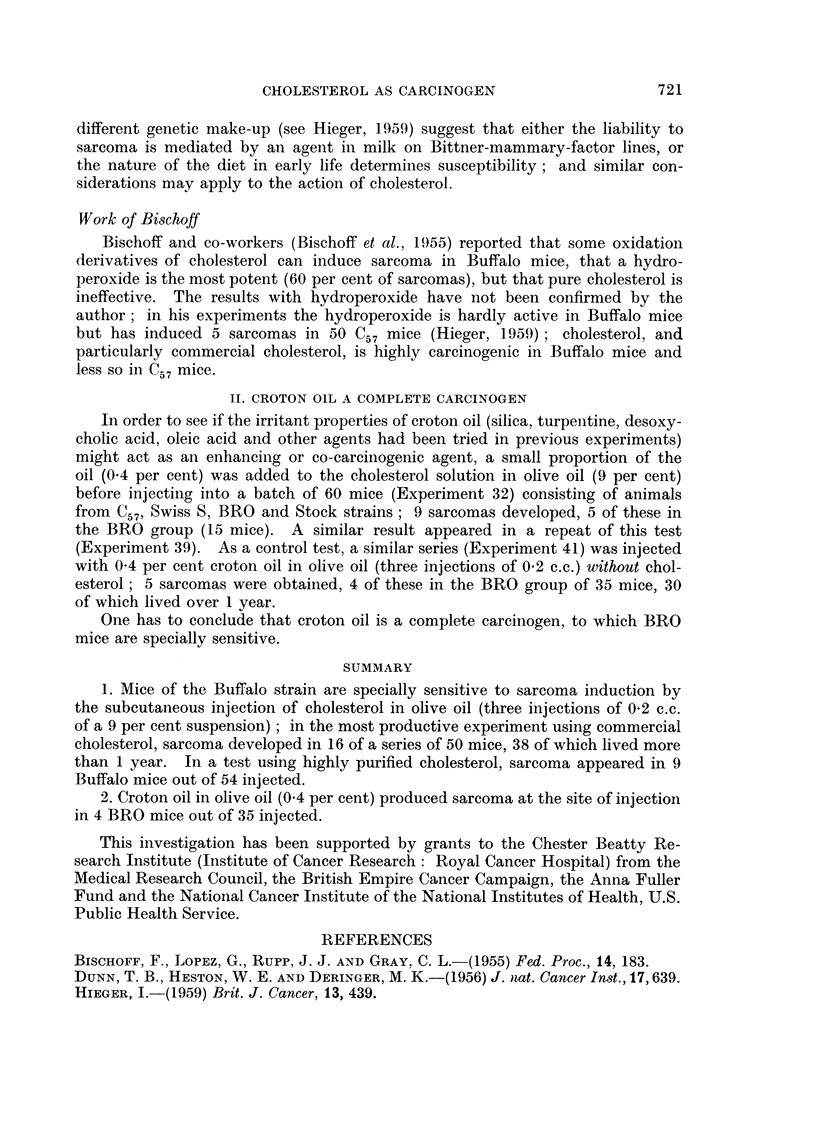# Cholesterol as Carcinogen

**DOI:** 10.1038/bjc.1962.83

**Published:** 1962-12

**Authors:** I. Hieger


### CHOLESTEROL AS CARCINOGEN

### I. Sarcoma Induction by Cholesterol in a Sensitive Strain of Mice

### II. Croton Oil a Complete Carcinogen

I. HIEGER

*From the Chester Beatty Research Institute, Royal Cancer Hospital, London, S.W.3*

Received for publication August 6, 1962

### I. SARCOMA INDUCTION BY CHOLESTEROL IN A SENSITIVE STRAIN OF MICE

Earlier papers in this series (see e.g. Hieger, 1959) have presented experimental results showing that :

(a) the carcinogenic factor in the unsaponifiable fraction of human tissue (liver, lung, kidney, muscle) is cholesterol ;
(b) commercial cholesterol injected subcutaneously induces sarcoma in mice and stringent purification of such cholesterol does not impair its potency ;
(c) different strains of mice and even different batches of the same pure strain show gross differences of susceptibility to carcinogenesis by cholesterol ;
(d) the solvents used for administering the cholesterol (e.g. olive oil) have a very low sarcoma-inducing potency (5 tumours in 1122 mice).

The first part of this paper deals with an explanation of (c), i.e. the differences in susceptibility to cholesterol.

The table shown below (Table I) summarises the results of experiments carried out on cholesterol as carcinogen subsequent to those described in the 1959 paper.

Where the "Code" (laboratory labelling) of an experiment carries an asterisk(*), the mice were housed in a room where hydrocarbon-carcinogens (e.g. benzopyrene) are used (in order to test the effects of contamination). All other experiments were carried out in a room free from hydrocarbon-carcinogens. It is obvious from the results that atmospheric contamination by powerful carcinogens does not carry any risk to experiments where cholesterol is being tested.

The experiments are entered in the table in the order in which they were carried out, that is to say, Experiment 1 was started then 2, then 3, etc. This arrangement is useful in demonstrating that no appreciable part of the carcinogenic potency of the active preparations can be attributed to possible variations in the composition of successive batches of the vehicle (olive oil) which is a commercial product, and therefore different samples (hospital dispensary quality) might well have differences in composition owing to seasonal variations or to geographical changes in the source of the olive crop. For example, Experiment 27 gave a high yield of tumours but Experiment 28 a very low yield ; since these experiments were started within a few weeks of each other the composition of the oil can hardly be invoked to explain differences of carcinogenic potency.

Unless otherwise stated the concentration of cholesterol was 9 per cent in olive oil ; in the first few experiments the preparations were made by dissolving 1 g. of cholesterol in 10 g. olive oil on the water bath, and 0·2 c.c. of the hot solution was injected in the mice subcutaneously three times at intervals of about three weeks. In the later, and great majority of the experiments, the preparations were made rather differently : 1 g. cholesterol and a little olive oil were thoroughly stirred into a paste with a glass rod, then the remainder of the 10 g. of olive oil was added and stirred well, the whole heated on the water bath till solution and rapidly cooled with stirring. A paste was thus obtained which had been heated for only a few minutes and could be injected cold through the usual No. 1 needle.

The results of the experiments in Table I show that :

(a) under the conditions existing in our laboratory in the last few years, mice of $C_{57}$, $C_{57} \times C_3H$, Swiss S, BALB/C and Stock strains when injected thrice with 0·2 c.c. of a 9 per cent suspension of commercial or pure cholesterol have shown, at the site of injection, an incidence of sarcoma which ranged between about 5 and 0 per cent ;

(b) the incidence is sometimes increased by enlarging the dose of cholesterol ; by giving the first injection when the mice are only 1 day old ; by crowding a number of mice in a large cage instead of housing them as is usual 5 to a cage ; in one or two experiments by substituting lard for olive oil ; or by adding egg powder to the diet :

(c) the yield of sarcomas is much more effectively increased by using mice of the Buffalo and the BRO strains, or by adding a small proportion of croton oil to the cholesterol preparation ; in the most productive experiment (No. 45), sarcomas developed in 16 Buffalo mice out of an initial lot of 50, of which 38 lived more than 1 year, the yield being thus more than 40 per cent ;

(d) in BRO mice sarcoma will develop at the site of injection of croton oil (0·4 per cent) in olive oil.

*Discussion*

The obvious conclusion to be drawn from Table I is that the genetic nature of the mouse is the decisive factor in determining susceptibility to carcinogenesis by cholesterol, or by croton oil ; but it is also necessary to explain why some batches of stock mice (i.e. mice of unknown ancestry, originally obtained from a dealer a decade or two ago and bred in the laboratory without regard to relationship at mating) and of $C_{57}$ strain mice can sometimes develop sarcoma in over 10 per cent of their number on injection with cholesterol, while other batches do so in only 2 per cent or are even resistant (0 per cent).

As a first step it might be suggested that the requirements for susceptibility to carcinogenesis by cholesterol, or by threshold doses of carcinogenic hydrocarbons (see Hieger, 1959, for a development of this theme) depend upon slight alterations in the genetic controlling system, alterations so slight that they can be found in different batches of what are alleged to be pure line strains of mice.

The studies of Dunn, Heston and Deringer (1956) on the incidence of spontaneous sarcoma in different groups of pure line mice suckled by foster-mothers of

TABLE I.—*Sarcoma Induction in Mice by Cholesterol*

| Experiment | Code | Preparation C = cholesterol Comm. = commercial | Strain of mice | Number of mice and survivors (months): Initial | After 12 m. | After 18 m. | After 24 m. | Sarcomas | % Incidence on initial number | Calculated on survivors at 1 year (%) | |
|---|---|---|---|---|---|---|---|---|---|---|---|
| 1 | 374 C | Pure C in olive oil (9%) | Stock | 60 | 34 | 14 | 0 | 1 | 2 | 3 | In all experiments, unless otherwise stated, each mouse received 3 injections of 0·2 c.c. of a 9% solution of C. |
| 2 | 376 E | Pure C in olive oil (9%) | Stock | 63 | 40 | 21 | 4 | 0 | — | — | — |
| 3 | Fenu* | Comm. C in olive oil | $C_{57}$ | 536 | — | — | — | 12 | 2·2 | — | — |
| 4 | Janu* | Comm. C in sesame oil | $C_{57}$ | 49 | 36 | 28 | 9 | 0 | — | — | — |
| 5 | Sibo* | Comm. C in olive oil + tristearin (12%) | Stock | 50 | 35 | 14 | 3 | 0 | — | — | — |
| 6 | Peti* | Comm. C in olive oil | $C_3H \times C_{57}$ | 65 | 62 | 49 | 30 | 1 | 2 | 2 | — |
| 7 | Xozu* | Comm. C in olive oil + oleic acid 9% | Stock, Swiss S | 50 | 32 | 2 | 2 | 0 | — | — | — |
| 8 | Zave* | Acetone-crystallised C in lard (13%) | Stock, Swiss | 50 | 41 | 18 | 6 | 1 | 2 | 2·5 | — |
| 9 | 415 R | Acetone-crystallised C in lard (13%) | Stock, Swiss | 50 | 32 | 23 | 6 | 1 | 2 | 3 | — |
| 10 | Bewi* | Acetone-crystallised C in lard (13%) | Stock, $C_{57}$ | 71 | 29 | 11 | 6 | 3 | 4 | 10 | (5 injections of 0·3 c.c. each. Egg powder in diet.) |
| 11 | Dixo* | Acetone-crystallised C in lard (13%) | Stock, $C_{57}$ | 50 | 25 | 14 | 8 | 4 | 8 | 16 | (3 injections of 0·75 c.c). |
| 12 | 377 F | Pure C in olive oil (30%) | Stock | 47 | 33 | 22 | 3 | 0 | — | — | — |
| 13 | 381 J | Pure C in olive oil 9% | $C_{57}$, Stock | 67 | 47 | 38 | 8 | 1 | 1·5 | 2 | - |
| 14 | 382 K | Pure C in sesame oil + 20% stearic acid | $C_{57}$, Stock | 59 | 38 | 19 | 7 | 0 | — | — | — |
| 15 | 383 L | Pure C in olive oil | $C_{57}$, Stock | 68 | 53 | 29 | 19 | 2 | 3 | 4 | (Mice also had 4 injections oestrone 70$\gamma$.) |
| 16 | 384 M | Pure C in olive oil | $C_{57}$ | 53 | 43 | 32 | 8 | 3 | 6 | 7 | — |
| 17 | 385 N | Comm. C in olive oil | $C_{57}$ | 69 | 56 | 26 | 21 | 3 | 4 | 6 | Egg powder in dt as in Experiment 10. |
| 18 | 387 P | Pure C in olive oil | $C_{57}$ | 80 | 67 | 60 | 28 | 1 | 1·3 | 1·5 | Young mice < 1 month of age. |
| 19 | 388 Q | Pure C in olive oil | $C_{57}$, Swiss S | 50 | 37 | 23 | 21 | 3 | 6 | 10 | Young mice, 1 day old. |

| | | | | | | | | | | |
|---|---|---|---|---|---|---|---|---|---|---|
| 20 | 389 R | Pure C in olive oil | Swiss S | 51 | 31 | 11 | 5 | 1 | 2 | 3 | — |
| 21 | 395 X, 396 Y | Comm. C in olive oil suspension 25% | Swiss, $C_3H \times C_{57}$ | 63 | 54 | 42 | 32 | 1 | 2 | — | — |
| 22 | 397 Z | Comm. C in olive oil 15% | $C_{57}$, Stock, $C_3H \times C_{57}$ | 61 | 52 | 37 | 23 | 3 | 5 | 6 | Mice were injected 6 times |
| 23 | 401 D | Pure C in olive oil | Stock, $C_{57}$ | 50 | 25 | 13 | 6 | 0 | — | — | — |
| 24 | 402 E | Comm. C in olive oil 15% | Stock, $C_{57}$ | 88 | 72 | 22 | 0 | 1 | 1·3 | — | Castrated mice. |
| 25 | 403 F | Comm. C in glyceryl-mono-oleate, 25% | Stock, $C_{57}$ | 54 | 34 | 22 | 7 | 1 | 2 | — | — |
| 26 | 405 H, 410 M | Comm. C in olive oil | Stock, $C_{57}$ | 80 | 44 | 36 | 17 | 5 | 6 | 12 | A " crowding " experiment, 20 mice per cage. |
| 27 | 407 J | Comm. C in olive oil | Buffalo | 51 | 36 | 32 | 7 | 12 | 24 | 33 | — |
| 28 | 409 L | Comm. C in olive oil | Stock, $C_3H \times C_{57}$ | 52 | 28 | 19 | 7 | 1 | 2 | 4 | Mice had 300 r $\gamma$-irradiation. |
| 29 | 411 N | Comm. C in olive oil + tristearin | Stock, Swiss S | 50 | 28 | 19 | 4 | 1 | 2 | 4 | — |
| 30 | 412 O | Comm. C in olive oil | Stock, Swiss S, BRO, $C_{57}$ | 47 | 30 | 12 | 1 | 4 | 8 | 13 | Mice when 1 day old received 0·01 c.c. turpentine. |
| 31 | 415 R | C (crystallised from acetone) in lard, 15% | Stock, BRO, Swiss S | 50 | 30 | 23 | 6 | 4 | 8 | 13 | — |
| 32 | 416 S | Comm. C in olive oil, 9% + 0·4% croton —oil | $C_{57}$, Stock, Swiss S, BRO | 60 | 52 | 24 | 4 | 9 | 15 | 17 | 5 of the 9 sarcomas appeared in the 15 BRO mice of which 11 lived > 1 year. |
| 33 | 418 U | Comm. C in olive oil | LAC | 62 | 38 | 28 | 7 | 0 | — | — | — |
| 34 | 419 V | C (crystallised from acetone) in olive oil | $C_{57}$ | 50 | 29 | 22 | 8 | 0 | — | — | Each mouse had 6 injections. |
| 35 | 421 X | Comm. C in olive oil | BALB/C | 43 | 31 | 22 | 14 | 2 | 5 | — | — |
| 36 | 425 B | Comm. C in olive oil | 101 | 32 | 16 | 3 | 1 | 0 | — | — | — |
| 37 | 427 D | Pure C in olive oil | Buffalo | 63 | 44 | 24 | 0 | 7 | 11 | 21 | — |
| 38 | 429 F | C (crystallised from acetone) in olive oil | BRO | 65 | 46 | 40 | — | 2 | 4 | — | — |
| 39 | 430 G | C (crystallised from acetone) in olive oil + 0·4% croton oil | Stock, BRO, $C_{57}$, 101 | 80 | 34 | 23 | — | 7 | 9 | 21 | 5 of the 7 sarcomas appeared in the 35 BRO mice, 23 of which lived > 1 year. |
| 40 | Migo* | C (crystallised from acetone) in olive oil + 0·4% croton oil | Stock, $C_{57}$, BRO | 61 | 34 | — | — | 3 | 5 | 10 | — |

TABLE I.—*Sarcoma Induction in Mice by Cholesterol*—(continued)

| Experiment | Code | Preparation C = cholesterol Comm. = commercial | Strain of mice | Number of mice and survivors (months): Initial | After 12 m. | After 18 m. | After 24 m. | Sarcomas | % Incidence on initial number | Calculated on survivors at 1 year (%) | |
|---|---|---|---|---|---|---|---|---|---|---|---|
| 41 | 431 H | NO cholesterol, 0·4% croton oil in olive oil | Stock BRO $C_{57}$ 101 | 71 | 47 | 33 | — | 5 | 7 | 10 | 4 of the 5 sarcomas were in the 35 BRO mice. |
| 42 | 433 J | Pure C in olive oil | BRO | 50 | 46 | 31 | — | 3 | 6 | 6 | — |
| 43 | 434 K | Pure C in olive oil | Buffalo | 54 | 46 | — | — | 9 | 17 | 20 | — |
| 44 | 435 L | C crystallised from acetone, in olive oil | BRO | 50 | 40 | — | — | 9 | 18 | 22 | — |
| 45 | 437 N | Comm. C in olive oil | Buffalo | 50 | 38 | — | — | 16 | 32 | 40 | Latent periods (months) 6, 6½, 7, 7, 7½, 9, 9, 10, 10, 10, 10½, 10½, 10½, 12½, 13, 14. |
| 46 | 428 E | NO cholesterol, control on olive oil alone | Buffalo | 60 | 41 | 19 | — | 0 | 0 | 0 | Control test on solvent. |

different genetic make-up (see Hieger, 1959) suggest that either the liability to sarcoma is mediated by an agent in milk on Bittner-mammary-factor lines, or the nature of the diet in early life determines susceptibility ; and similar considerations may apply to the action of cholesterol.

*Work of Bischoff*

Bischoff and co-workers (Bischoff *et al.*, 1955) reported that some oxidation derivatives of cholesterol can induce sarcoma in Buffalo mice, that a hydroperoxide is the most potent (60 per cent of sarcomas), but that pure cholesterol is ineffective. The results with hydroperoxide have not been confirmed by the author ; in his experiments the hydroperoxide is hardly active in Buffalo mice but has induced 5 sarcomas in 50 $C_{57}$ mice (Hieger, 1959) ; cholesterol, and particularly commercial cholesterol, is highly carcinogenic in Buffalo mice and less so in $C_{57}$ mice.

### II. CROTON OIL A COMPLETE CARCINOGEN

In order to see if the irritant properties of croton oil (silica, turpentine, desoxycholic acid, oleic acid and other agents had been tried in previous experiments) might act as an enhancing or co-carcinogenic agent, a small proportion of the oil (0·4 per cent) was added to the cholesterol solution in olive oil (9 per cent) before injecting into a batch of 60 mice (Experiment 32) consisting of animals from $C_{57}$, Swiss S, BRO and Stock strains ; 9 sarcomas developed, 5 of these in the BRO group (15 mice). A similar result appeared in a repeat of this test (Experiment 39). As a control test, a similar series (Experiment 41) was injected with 0·4 per cent croton oil in olive oil (three injections of 0·2 c.c.) *without* cholesterol ; 5 sarcomas were obtained, 4 of these in the BRO group of 35 mice, 30 of which lived over 1 year.

One has to conclude that croton oil is a complete carcinogen, to which BRO mice are specially sensitive.

### SUMMARY

1. Mice of the Buffalo strain are specially sensitive to sarcoma induction by the subcutaneous injection of cholesterol in olive oil (three injections of 0·2 c.c. of a 9 per cent suspension) ; in the most productive experiment using commercial cholesterol, sarcoma developed in 16 of a series of 50 mice, 38 of which lived more than 1 year. In a test using highly purified cholesterol, sarcoma appeared in 9 Buffalo mice out of 54 injected.

2. Croton oil in olive oil (0·4 per cent) produced sarcoma at the site of injection in 4 BRO mice out of 35 injected.

This investigation has been supported by grants to the Chester Beatty Research Institute (Institute of Cancer Research : Royal Cancer Hospital) from the Medical Research Council, the British Empire Cancer Campaign, the Anna Fuller Fund and the National Cancer Institute of the National Institutes of Health, U.S. Public Health Service.

### REFERENCES

BISCHOFF, F., LOPEZ, G., RUPP, J. J. AND GRAY, C. L.—(1955) *Fed. Proc.*, **14**, 183.
DUNN, T. B., HESTON, W. E. AND DERINGER, M. K.—(1956) *J. nat. Cancer Inst.*, **17**, 639.
HIEGER, I.—(1959) *Brit. J. Cancer*, **13**, 439.